# Trimester- and Assay-Specific Thyroid Reference Intervals for Pregnant Women in China

**DOI:** 10.1155/2016/3754213

**Published:** 2016-03-21

**Authors:** Jinfang Xing, Enwu Yuan, Jing Li, Yuchao Zhang, Xiangying Meng, Xia Zhang, Shouhua Rong, Zhongxing Lv, Yuan Tian, Liting Jia

**Affiliations:** Clinical Laboratory Department, The Third Affiliated Hospital of Zhengzhou University, No. 7 Front Kangfu Street, Er'qi Distric, Zhengzhou 450052, China

## Abstract

*Objective*. The guidelines of the American Thyroid Association (ATA) recommend an upper limit reference interval (RI) of thyroid stimulating hormone (TSH) of 2.5 mIU/L in the first trimester of pregnancy and 3.0 mIU/L in subsequent trimesters, but some reported ranges in China are significantly higher. Our study aimed to establish trimester- and assay-specific RIs for thyroid hormones in normal pregnant Chinese women.* Methods*. In this cross-sectional study, 2540 women with normal pregnancies (first trimester, *n* = 398; second trimester, *n* = 797; third trimester, *n* = 1345) and 237 healthy nonpregnant control subjects were recruited. Serum TSH, free thyroxin (FT4), thyroid peroxidase antibody (TPOAb), and thyroglobulin antibody (TgAb) levels were determined by automated chemiluminescence with an Immulite 2000 system (Siemens, Erlangen, Germany). After outliers were excluded, the 2.5–97.5th percentiles were used to define the RIs.* Results*. The RIs of thyroid function in the first, second, and third trimesters of pregnancy and in nonpregnant controls were 0.07–3.96, 0.27–4.53, 0.48–5.40, and 0.69–5.78 mIU/L for TSH and 9.16–18.12, 8.67–16.21, 7.80–13.90, and 8.24–16.61 pmol/L for FT4, respectively.* Conclusion*. The trimester- and assay-specific RIs of thyroid function during pregnancy differed between trimesters, which suggests that it is advisable to detect and avoid misclassification of thyroid dysfunction during pregnancy for women in Henan, China.

## 1. Introduction

Maternal thyroid hormones are known to contribute to fetal development. Thyroid dysfunction is coupled to adverse outcomes in both the mother and the fetus and is particularly associated with the cognitive and neurological development of the fetus [1, 2]. Women are more susceptible to developing thyroid disease later in life if they exhibit thyroid dysfunction or test positive for antibodies during pregnancy [3]. The early and appropriate detection of thyroid dysfunction and timely interventions that improve maternal-fetal prognosis require reliable gestational specific reference intervals (RIs) of the thyroid hormones.

During pregnancy, hormonal changes and metabolic demands complicate the diagnosis of thyroid dysfunction. Moreover, many established thyroid RIs during pregnancy differ by assay, ethnicity, and region making it difficult to extrapolate values for all women and areas [4–11]. Thus, the use of trimester- and assay-specific RIs in pregnancy is recommended by clinical guidelines [12, 13]. The aim of this study is the establishment of trimester- and assay-specific RIs of thyroid stimulating hormone (TSH) and free thyroxin (FT4) for pregnancy in an iodine-sufficient area of Henan, China.

## 2. Materials and Methods

### 2.1. Study Population

A total of 3314 women who consumed iodized salt were initially enrolled from the outpatient and inpatient departments of the Third Affiliated Hospital of Zhengzhou University from January 2013 until January 2014. The exclusion criteria consisted of a history of thyroid disease or goiter, use of medications that may affect thyroid function, any other chronic diseases, patients positive for thyroid peroxidase and/or thyroglobulin antibodies (TPOAb > 35 IU/mL, TgAb > 40 IU/mL, chemiluminescence assay), pregnancy-associated complications, or twin pregnancy. A total of 501 women were excluded due to the exclusion criteria, 36 were excluded as outliers, and the remaining 2777 women were assigned to four groups according to the number of weeks of gestation: *T*1 = 1–12 weeks, *T*2 = 13–27 weeks, *T*3 = 28–40 weeks, and nonpregnant women. The median gestational ages (range) at the first, second, and third trimesters were 12.0 (4.3–12.9), 17.6 (13.0–27.9), and 37.6 (28.0–41.9) weeks, respectively. The mean ages (range) for women in their first, second, and third trimesters and nonpregnant women were 29.1 ± 4.8 (16–44), 28.9 ± 4.6 (18–47), 28.9 ± 5.1 (17–48), and 29.1 ± 4.7 (19–43) years, respectively, with no significant difference. This study was approved by the Ethics Committee at the Third Affiliated Hospital of Zhengzhou University. Informed consent was obtained from all subjects.

### 2.2. Methods

Fasting blood samples were collected from all the subjects in a sitting position in the morning and centrifuged at 4000 rpm for 10 min within 1 hour. Subsequently, the serum TSH, FT4, TPOAb, and TgAb levels were assayed using automated chemiluminescence within 2 hours with an Immulite 2000 system and Siemens kits (Siemens Healthcare Diagnostics Products Limited, Munich, Germany) according to the manufacturer's instructions. The functional sensitivities of serum TSH, FT4, TPOAb, and TgAb were 0.004 mIU/L, 3.99 pmol/L, 5.0 IU/mL, and 2.2 IU/mL, respectively. Daily quality control was performed via a third party (Bio-Rad). All measurements were controlled.

### 2.3. Statistical Analysis

The RIs were calculated according to the guidelines of the Clinical and Laboratory Standards Institute. Box plots were used to identify the possible outliers, which were excluded when the *D*/*R* ratio was greater than 1/3. For TSH and FT4 analyses, the medians and 2.5–97.5th percentiles were defined for all reference subjects. In comparisons between the study groups, age was assessed using a one-way ANOVA followed by a Bonferroni correction, while the TSH and FT4 levels were assessed using Kruskal-Wallis one-way ANOVA. All statistical analyses were performed with SPSS version 17.0 software (SPSS Inc., Chicago, IL, USA). *P* values < 0.05 were considered statistically significant.

## 3. Results

### 3.1. TSH and FT4 RIs in Pregnant and Nonpregnant Women

We identified 36 patients as outliers using box plots, and data from these patients were excluded from the analysis. The TSH RIs in the first, second, and third trimesters of pregnancy were 0.07–3.96, 0.27–4.53, and 0.48–5.40 mIU/L, respectively, while the TSH RI was 0.69–5.78 mIU/L in the nonpregnant group. The FT4 RIs in the first, second, and third trimesters were 9.16–18.12, 8.67–16.21, and 7.80–13.90 pmol/L, respectively, but the RI was 8.24–16.61 pmol/L in nonpregnant women (Table 1).

**Figure 1:**
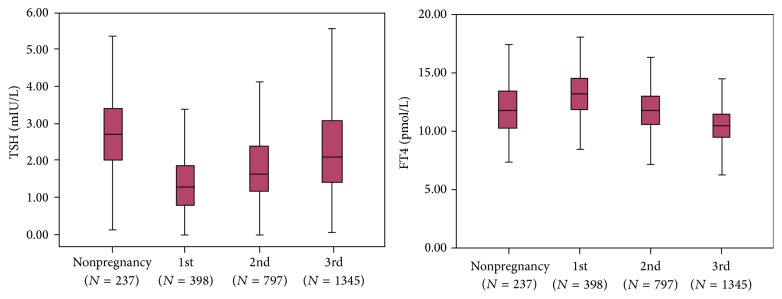
The trends of the TSH and FT4 levels during pregnancy and in nonpregnant women. The central box represents values from the lower to the upper quartile. The middle line represents the median, and the bars represent the 2.5th and 97.5th percentiles.

The TSH level exhibited a significantly (*P* < 0.001) increasing trend from the first to the third trimester and remained lower than the prepregnancy levels. FT4 level showed a significantly decreasing trend with increasing gestational age (*P* < 0.001) and was the lowest in the third trimester (Figure 1).

## 4. Discussion

During pregnancy, metabolic demands increase, and hormonal changes result in alterations of the pituitary-thyroid axis. The negative feedback of TSH decreases due to TSH receptor activation by human chorionic gonadotropin (HCG), which shares alpha-subunits and considerable homology with the beta-subunit of TSH. The TSH level peaks at 10–12 weeks of gestation, while triiodothyronine (T3) and thyroxine (T4) levels increase up to 50% with an accompanying increase in thyroxin binding globulin (TBG) levels. The clearance of iodine in the kidneys is enhanced during pregnancy [12, 14]. Thus, it is inappropriate to assess thyroid function by T3 and T4. In this case, TSH and FT4 are the most commonly used indicators of thyroid dysfunction.

Currently, in China, different trimester-specific ranges which were provided by the manufacturers were used. However, these ranges varied significantly and were higher than 2.5 mIU/L, which was recommended by the American Thyroid Association (ATA). Thus, it was suggested that each region or even each laboratory should establish its own trimester-specific RIs by China [15]. Establishing trimester-specific reference intervals in each population is essential for accurate assessment of thyroid function. The exclusion criteria were strictly applied in our study. This study aimed to provide trimester- and assay-specific RIs of TSH and FT4 in pregnant women in Henan, China, in an iodine-sufficient area. These population-specific and method-specific reference intervals will be useful for screening China pregnant women for thyroid disease.

In our study, the lower limits of TSH RIs were similar to those of the ATA guidelines and other studies. However, the upper limits of TSH were higher than those observed in Caucasian populations and comparable to those in Asian populations (Table 2). This confirmed the results of La'ulu and Roberts [16], who found that Asians have increased levels of TSH. In the three studies that used the Immulite 2000 assay for Indian, Korean, and Chinese (our study) subjects, the upper TSH limit was higher than in other studies, which could be a consequence of the combined effects of ethnicity and methodology. Previous studies in China have shown similar RIs of TSH, regardless of the assay used [4, 5, 7, 8, 17].

The RIs for FT4 in our study were comparable to those of other studies (Table 2) with mild differences, possibly resulting from different measurement techniques. van Deventer and Soldin [18, 19] stated that the optimal method to assess FT4 during pregnancy is liquid chromatography/tandem mass spectrometry (LC/MS/MS) because most immunoassays have a significant method-specific bias related to the binding proteins. However, LC/MS/MS is not available in most clinical laboratories, which suggests that standardizing thyroid function tests should be a major priority [20, 21].

La'ulu and Roberts [22] found higher RIs in Asian populations. In our study, the rates of positive TPOAbs and TgAbs were 8.20% (270/3292) and 5.04% (166/3292), and the double positive rate was 2.58% (85/3292) when thyroid disease or goiter was excluded. In the TPOAb- and/or TgAb-positive individuals, 74.07% (260/351) were pregnant, and 25.93% (91/351) were not pregnant. However, we only enrolled antibody-negative women for RI determination.

Thus, other factors such as the exclusion criteria, study population characteristics, method of RI estimation, and iodine status could contribute to this discrepancy. Therefore, a direct comparison was not appropriate. It is essential to establish trimester-specific and assay-specific RIs in Asian countries to accurately assess thyroid function.

Our study has several limitations. First, thyroid function could not be measured consecutively in each individual, and interindividual variation could exist. Subsequently, the iodine status was not evaluated. However, the daily consumption of iodized salt has been mandatory in China for many years; therefore, we assumed that all enrolled participants ingested sufficient amounts of iodine. In conclusion, we established RIs for TSH and FT4 in pregnant Chinese women. These RIs will be useful for screening for thyroid diseases in pregnant Chinese women.

## Figures and Tables

**Table 1:** Reference intervals for TSH and FT4 in each trimester of pregnancy and in nonpregnant women.

Groups	*N*	TSH (mIU/L)			FT4 (pmol/L)		
		Median	2.5th percentile	97.5th percentile	Median	2.5th percentile	97.5th percentile
Nonpregnant	237	2.69	0.69	5.78	11.60	8.24	16.61
*Pregnancy*							
First trimester	398	1.30	0.07	3.96	13.10	9.16	18.12
Second trimester	797	1.64	0.27	4.53	11.70	8.67	16.21
Third trimester	1345	2.10	0.48	5.40	10.30	7.80	13.90

**Table 2:** Summary of trimester-specific RIs for TSH and FT4.

Assays and references	Country	Enrolled number	Excluded TPOAb, TgAb	Percentile used		Reference interval		
						First trimester	Second trimester	Third trimester
*Immulite 2000 *								
Lambert-Messerlian et al., 2008 [9]	USA	9,562	Yes	5–95	TSH	0.12–2.68	0.35–2.77	
Karakosta et al., 2011 [11]	Greece	425	Yes	2.5–97.5	TSH FT4	0.05–2.53 12.36–20.59	0.18–2.73 10.81–18.53	
Present study	China	3314	Yes	2.5–97.5	TSH FT4	0.07–3.96 9.16–18.12	0.27–4.53 8.67–16.21	0.48–5.40 7.80–13.90
*Abbott Architect*								
Stricker et al., 2007 [23]	Switzerland	2,272	Yes	2.5–97.5	TSH FT4	0.088–2.829 10.53–18.28	0.199–2.792 9.53–15.68	0.307–2.903 8.63–13.61
Shen et al., 2014 [17]	China	1409	Yes	2.5–97.5	TSH FT4	0.16–3.78 10.9–17.7	0.34–3.51 9.3–15.2	0.34–4.32 7.9–14.1
*Roche Elecsys*								
Marwaha et al., 2008 [24]	India	541	Yes	5–95	TSH FT4	0.6–5.0 12–19.45	0.44–5.78 9.48–19.58	0.74–5.7 11.3–17.71
Yu et al., 2010 [7]	China	538	Yes	2.5–97.5	TSH FT4	0.02–3.65 11.85–21.51	0.36–3.46 9.45–16.26	0.44–5.04 9.3–17.14
Moon et al., 2015 [10]	Korea	769	Yes	2.5–97.5	TSH FT4	0.01–4.10 10.68–21.24	0.01–4.26 9.13–15.70	0.15–4.57 8.37–14.54
Zhang et al., 2015 [8]	China	3507	Yes	2.5–97.5	TSH FT4	0.06–3.13 8.72–15.22	0.07–4.13 7.10–13.55	0.15–5.02 6.16–12.03
Wang et al., 2011 [5]	China	1455	TPOAb	2.5–97.5	TSH FT4	0.19–3.54 12.01–24.62	0.38–3.29 9.53–16.91	0.51–5.43 9.37–17.14
*Advia Centaur*								
Yan et al., 2011 [4]	China	505	Yes	2.5–97.5	TSH FT4	0.03–4.51 11.8–21.0	0.05–4.50 10.6–17.6	0.47–4.54 9.2–16.7

The units of TSH and FT4 in published studies were mIU/L and pmol/L, and the units were converted from the original data when necessary.
